# Early outcomes of a triple-branched stent graft implantation in elderly patients with acute type a aortic dissection

**DOI:** 10.1186/s12872-023-03513-3

**Published:** 2023-10-31

**Authors:** Yi Dong, Xuefei Wang, Liangwan Chen, Qingsong Wu, Qianzhen Li

**Affiliations:** 1 Department of Cardiovascular Surgery, Union Hospital, Fujian Medical University, Fuzhou, Fujian China; 2 Key Laboratory of Cardio-Thoracic Surgery, Fujian Medical University, Fujian Province University, Fuzhou, Fujian China; 3 Fujian Medical university, Fuzhou, Fujian China; 4 Fujian Key Laboratory of Vascular Aging, Fujian Medical University), Fuzhou, Fujian China

**Keywords:** Acute type a aortic dissection, Triple-branched stent graft, Aortic arch, Elderly patients

## Abstract

**Purpose:**

Older patients with acute type A aortic dissection (ATAAD) have higher risk of mortality than that of younger patients when a total arch reconstruction (TAR) is required. Triple-branched stent graft (TBSG) implantation is a novel technique for TAR. However, early outcomes of a TBSG implantation in older patients have not been reported. This study aimed to evaluate the early outcomes of the TBSG technique in older patients with ATAAD.

**Methods:**

From February 2015 to December 2020, 640 patients who simultaneously underwent an emergent open aortic surgery and TBSG implantation for ATAAD were enrolled in this study. They were categorized into the younger (age ≤ 70 years old, n = 573) and older groups (age > 70 years, n = 67). Clinical data of all patients were retrospectively reviewed.

**Result:**

The mean ages of the patients in the younger and older groups were 45.3 ± 9.6 years old and 73.5 ± 3.0 years old, respectively. Preoperative characteristics were similar between the two groups, except for weight and incidence of moderate or greater aortic regurgitation, which were lower in the older group than those in the younger group. Surgical procedure and duration (i.e., duration for cardiopulmonary bypass, aortic cross-clamp, selected cerebral perfusion, and total circulation arrest) were comparable between the two groups (p > 0.05). Patients in the older group had higher incidence of dialysis for acute kidney injury and longer ICU stay compared with those in the younger group. However, the incidences of 30-day mortality (5.1% in younger group vs. 7.5% in older group, p = 0.407) and other major complications (i.e., neurological adverse events) were similar between the two groups.

**Conclusion:**

TBSG implantation for ATAAD resulted in an acceptable mortality rate in patients above 70 years old, thus, it could be a feasible surgical procedure to perform in older patients with ATAAD when a TAR is required.

## Introduction

Acute type A aortic dissection (ATAAD) is a fatal disease that commonly requires emergent surgery. Total arch reconstruction (TAR) might be necessary when the aortic arch is severely involved. Due to the need of a circulatory arrest, aortic arch surgery is associated with higher risks of mortality and complications (i.e., cerebrovascular events), especially in older patients. Chung et al. [1] reported that, as age increases, patients experience elevated rates of mortality and major complications following an aortic arch surgery. Therefore, some surgeons would prefer to perform a hemiarch repair instead of a TAR in older patients. However, the residual dissection following an aortic arch surgery can lead to risks of rupture or reoperation [2–5]. Thus, patients may benefit from undergoing a TAR from the perspective of long-term outcomes although this procedure remains a challenge for some cardiac surgeons. We previously introduced a triple-branched stent graft (TBSG) technique for TAR [2–7]. This novel technique could reduce the number of anastomoses in the branched vessels and difficulty of distal arch anastomosis, leading to shorter duration of cerebral arrest and lower incidence of blood oozing, which theoretically could decrease the incidences of mortality and complications. However, the outcomes of a TBSG implantation in older patients with ATAAD have not been reported. The present study aimed to evaluate the early outcomes of the TBSG technique in older patients with ATAAD.

## Patients and methods

### Patients’ selection

From February 2015 to December 2020, 1,022 patients were admitted to our center for ATAAD. The diagnosis of ATAAD was confirmed via computed tomography angiography. An acute aortic dissection was defined as a dissection occurring within two weeks of symptom onset. Patients who simultaneously underwent an emergent open aortic surgery and TBSG implantation were enrolled in this study. The indication for TBSG implantation were aortic arch involvement that required a TAR (i.e., severe arch involvement or dissection entry located on aortic arch). Exclusion criteria included elective or chronic cases, retrograde aortic dissection for descending aortic dissection, aortic aneurysm, abnormal arch anatomy which was unsuitable for a TBSG implantation (i.e., bovine arch, aberrant right subclavian artery, and distance between the right subclavian artery orifice and innominate artery of < 35 mm). The study protocol was reviewed and approved by the institutional review board of the Union Hospital of Fujian Medical University in Fuzhou, China (No. 2,015,083). All patients were immediately brought to the operation room as soon as the diagnosis of ATAAD was confirmed, and informed consent was obtained from the patient’s family members.

### Triple-branched stent graft

The TBSG used in the present study was designed by Dr Chen and manufactured by Yuhengjia Sci Tech Corp Ltd, Beijing, China [4, 5]. The details of the surgical procedure have been previously described. The TBSG consists of a Dacron-covered self-expandable nitinol stent at the distal side, which functions as an “elephant trunk”, main graft without a stent at the proximal side, and three sidearm grafts. The main graft and three sidearm grafts were individually mounted on four small catheters and restrained using four silk strings. The sizes of the sidearm grafts and distal stented graft were determined based off the diameter of the supra-arch vessels and proximal descending aorta, respectively.

### Surgical procedure

The surgical procedure has been described in our previous studies [4, 5]. All procedures were performed with patients under general anesthesia. After a median sternotomy was performed, a cardiopulmonary bypass (CPB) was established. When the nasopharyngeal temperature reached 32℃, the ascending aorta was cross clamped and longitudinally incised, and cardioplegia was subsequently administered. Aortic root procedure was performed, if necessary, followed by an ascending aorta replacement. After the nasopharyngeal temperature reached 25–28℃, circulatory arrest began, and selected cerebral perfusion (SCP) was established through the right axillary cannula (flow rate, 8–10 mL·kg^− 1^·min^− 1^). The cross-clamp was removed, and the front wall of the aortic arch was transversely incised, after which the three arch vessel orifices and true lumen of the proximal descending aorta were clearly identified. The distal portion of the main graft was inserted into the true lumen of the descending aorta, and each sidearm graft was positioned inside the corresponding aortic arch vessel. The grafts were deployed by withdrawing the restraining strings. Two stitches were individually placed through the first two sidearm grafts and native aortic arch vessel wall. A cannula was placed into the left common carotid artery through the orifice of its sidearm graft for bilateral SCP (flow rate, 8–10 mL·kg-1·min-1). The transverse incision of the aortic arch was then closed using a continuous suture attached to the main graft of the TBSG. The proximal side of the aortic arch was connected to the distal prosthesis graft. After the air was flushed out, systemic perfusion resumed, and the patient was rewarmed. The patients were sent to the intensive care unit (ICU) after the surgery.

### Data collection and analysis

Clinical data of all patients were collected and retrospectively reviewed. Baseline characteristics, intraoperative data, and in-hospital results were summarized using descriptive statistics. Continuous variables were expressed as mean ± standard deviation and compared using t-test or Wilcoxon rank-sum test, as appropriate. Categorical variables were expressed as frequencies (%) and compared using Pearson’s χ2 test or Fisher’s exact test, as appropriate. Survival analyses were performed using the Kaplan-Meier method and log-rank test was used to calculate the P value. Statistical significance was set at p < 0.05.

## Results

A total of 640 patients were enrolled in the study. The characteristics of all patients are summarized in Table 1. The patients were categorized into groups according to their age, 573 patients were in the younger group (age ≤ 70 years old) and 67 patients were in the older group (age > 70 years). The mean ages of patients in the younger and older groups were 45.3 ± 9.6 years old and 73.5 ± 3.0 years old, respectively. The mean weight of patients in the older group was lower than that of the younger group. The incidence of diabetes in the younger group were lower than that of the older group (9.2% vs. 16.4%, p = 0.064), but the difference was not statistically significant. The younger group had higher incidence of moderate or severe aortic regurgitation than that of the older group (30.7% vs. 17.9%, p < 0.05). The incidences of hypertension, preoperative stroke, chronic lung disease, renal dysfunction, peripheral vascular disease, Marfan syndrome, prior cardiac surgery, and malperfusion syndrome were not significant different between these two groups. In addition, no significant differences were observed between the two groups for left ventricular ejection fraction and end-diastolic dimension detected by echocardiography.

**Table 1:** The preoperative characters of patients underwent TBSG implantation

Demographics	Total (n = 640)	Younger group (n = 573)	Older group (n = 67)	P value
Age, y	52.9 ± 11.3	45.3 ± 9.6	73.5 ± 3.0	< 0.001
Weight, kg	72.3 ± 14.6	76.5 ± 17.3	61.4 ± 9.9	< 0.001
Male, n (%)	491(76.7)	446 (77.8)	45 (67.2)	0.051
Hypertension, n (%)	521(81.5)	462 (80.6)	59 (88.1)	0.139
Diabetes, n (%)	64(10.0)	53 (9.2)	11 (16.4)	0.064
Preoperative stroke, n (%)	35 (5.5)	29 (5.1)	6 (0.9)	0.185
Chronic lung disease (moderate or greater), n (%)	42 (6.6)	35 (6.1)	7(10.4)	0.185
Renal dysfunction, n (%)	86 (13.4)	77 (13.4)	9 (13.4)	0.999
Peripheral vascular disease, n (%)	46 (7.2)	40 (7.0)	6 (0.9)	0.794
Marfan Syndrome, n (%)	38 (6.8)	38 (6.6)	0	N/A
Prior cardiac surgery, n (%)	43(6.7)	42 (7.3)	1 (1.5)	0.282
Moderate AR or greater, n (%)	178(27.9)	176 (30.7)	12 (17.9)	0.029
LVEF, %	62.8 ± 8.1	62.7 ± 9.2	61.9 ± 8.1	0.619
LVED, mm	48.5 ± 7.9	48.8 ± 8.4	49.0 ± 7.8	0.877
Malperfusion syndromes, n (%)				
Cerebral	32 (5.0)	29 (5.1)	3 (4.5)	0.951
Myocardial	26(4.1)	23 (4.0)	3 (4.5)	0.885
Renal	74(11.5)	68 (11.9)	6 (9.0)	0.481
Lower extremity	29(4.5)	25 (4.4)	4 (6.0)	0.773

TBSG: triple branched stent graft; LVEF: left ventricular ejected fraction; LVED: left ventricular end diastolic diameter

Surgical data of the patients are shown in Table 2. Patients in the two groups received comparable aortic valve or root procedure, coronary artery bypassing graft, and mitral surgery. The mean durations for CPB, aortic cross-clamp, SCP, and total circulation arrest in the younger group were 145.9 ± 36.3 min, 53.8 ± 22.5 min, 12.2 ± 4.5 min, and 4.6 ± 3.0 min, respectively, which were comparable with those in the older group (139.5 ± 26.4 min, 47.9 ± 15.7 min, 11.7 ± 4. 7 min, and 3.7 ± 23 min, p > 0.05, respectively).

**Table 2:** The surgical data of patients underwent TBSG implantation

Demographics	Total (n = 640)	Younger group (n = 573)	Older group (n = 67)	P value
Aortic valve or root surgery, n (%)	562 (87.8)	503 (87.8)	59 (88.1)	0.949
Aortic valve replacement	116 (18.1)	101 (17.6)	15 (22.4)	0.338
Bentall procedure	115 (18.0)	101 (17.6)	14 (20.9)	0.51
Reconstruction of the sinus of Vaslava	300 (46.9)	272 (47.5)	28 (41.8)	0.378
Valve-sparing root replacement	31 (4.8)	29 (5.1)	2 (3.0)	0.654
Comitant surgery				
CABG, n (%)	22 (3.4)	20 (3.5)	2 (3.0)	0.889
Mitral surgery, n (%)	15 (2.3)	14 (2.4)	1 (1.5)	0.952
CPB time, min	145.9 ± 35.6	145.9 ± 36.3	139.5 ± 26.4	0.343
Crossclamp time, min	52.4 ± 20.2	53.8 ± 22.5	47.9 ± 15.7	0.147
Selected cerebral perfusion time, min	12.0 ± 4.5	12.2 ± 4.5	11.7 ± 4.7	0.658
Circulation arrest time, min	4.3 ± 2.8	4.6 ± 3.0	3.7 ± 23	0.111

TBSG: triple branched stent graft; CABG: coronary artery bypass grafting; CPB: cardiopulmonary bypass

**Fig. 1:**
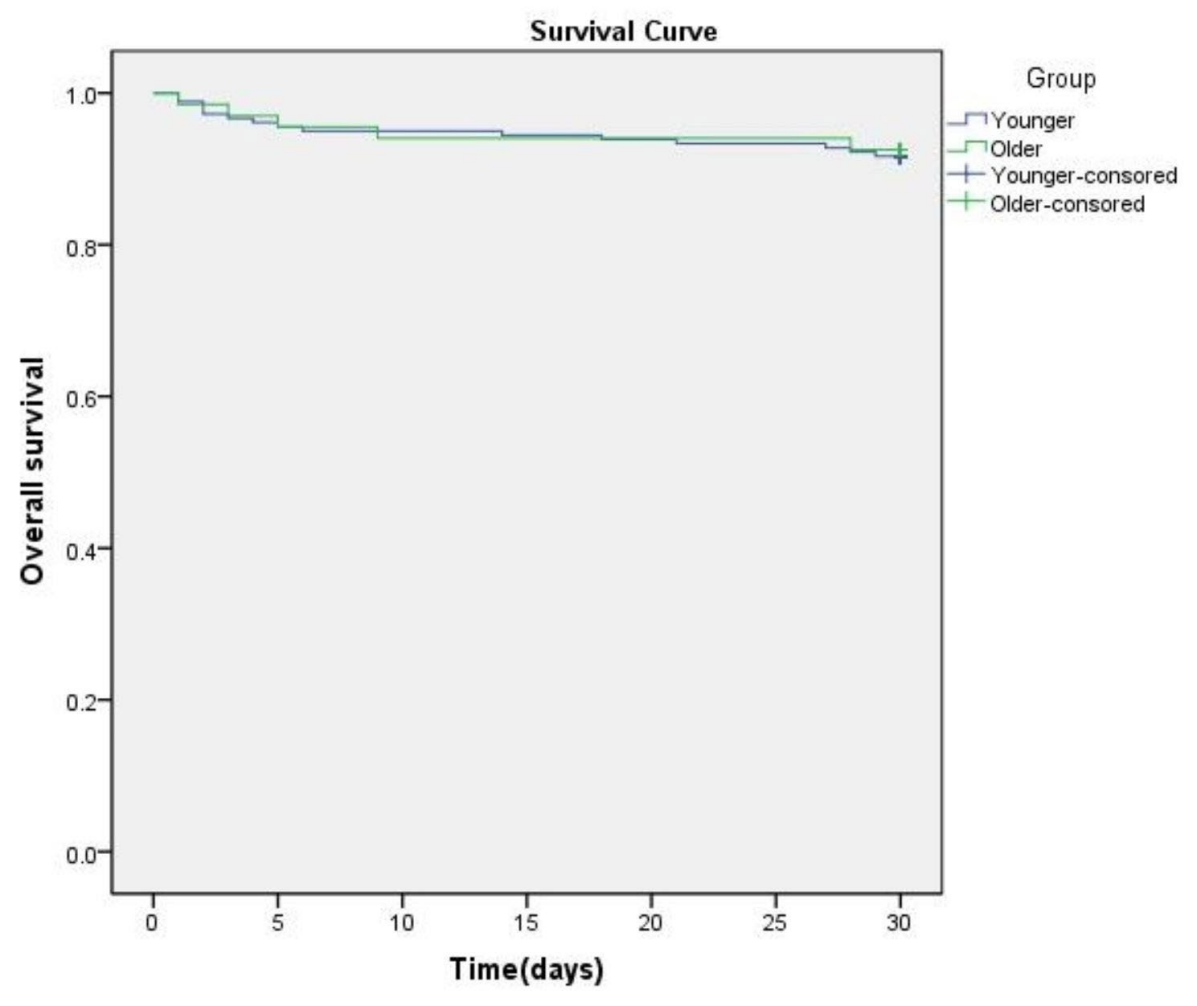
Survival curves for 30-day mortality in younger and older group

The total drainage volume after surgery (532.9 ± 341.3 mL in younger group vs. 514.7 ± 276.7 mL in older group, p = 0.675) and ventilation time (18.7 ± 7.4 h in younger group vs. 20.3 ± 10.8 h in older group, p = 0.11) were comparable in the two groups. Furthermore, the incidences of cardiac reoperation, myocardial infarction, sudden cardiac arrest, neurologic dysfunction, pneumonia, acute respiratory distress syndrome, acute kidney injury, gastrointestinal hemorrhage, sepsis, and sternal infection or dehiscence were similar between the two groups. Patients in the older group had higher incidence of dialysis for acute kidney injury compared with that of the younger group (26.9% vs. 15.2%, p = 0.015), which may result in prolonged ICU stay (67.5 ± 22.0 h vs. 87.8 ± 33.1 h, p < 0.05). However, the incidence of 30-day mortality (5.1% in younger group vs. 7.5% in older group, p = 0.407), the survival curves are shown in Fig. 1, and length of hospital stay (18.2 ± 11.9 days in younger group vs. 19.6 ± 13.6 days in older group, p = 0.786) were comparable between the two groups.(Table 3).

**Table 3:** The postoperative data of patients underwent TBSG implantation

Demographics	Total (n = 640)	Younger group (n = 573)	Older group (n = 67)	P value
The total drainage volume, ml	541.5 ± 356.6	532.9 ± 341.3	514.7 ± 276.7	0.675
Ventilation time, h	19.0 ± 10.2	18.7 ± 7.4	20.3 ± 10.8	0.11
ICU stay, h	70.4 ± 30.7	67.5 ± 22.0	87.8 ± 33.1	0.028
Hospital stay, d	18.3 ± 14.9	18.2 ± 11.9	19.6 ± 13.6	0.786
Cardiac reoperation, n (%)	9 (1.4)	8 (1.4)	1 (1.5)	0.628
Myocardial infarction, n (%)	8 (1.3)	7 (1.2)	1 (1.5)	0.695
Sudden Cardiac arrest, n (%)	18 (2.8)	16 (2.8)	2 (3.0)	0.764
Neurologic dysfunction, n (%)				
Temporary	20 (3.1)	18 (3.1)	2 (3.0)	0.763
Permanent	13 (2)	11 (1.9)	2 (3.0)	0.899
Pneumonia, n (%)	430(67.2)	383 (66.8)	47 (70.1)	0.585
ARDS, n (%)	16 (2.5)	14 (2.4)	2 (3.0)	0.885
Tracheotomy, n (%)	56 (8.8)	49 (8.6)	7 (10.4)	0.603
AKI, n (%)	177(27.7)	152 (26.5)	25 (37.3)	0.062
AKI required dialysis, n (%)	105(16.4)	87 (15.2)	18 (26.9)	0.015
Gastrointestinal hemorrhage, n (%)	14 (2.2)	12 (2.1)	2 (3.0)	0.976
Sepsis, n (%)	54(8.5)	47 (8.2)	7 (10.4)	0.532
Sternal infection or dehiscence, n (%)	4 (0.6)	3 (0.5)	1 (1.5)	0.894
30-d mortality, n (%)	34(5.3)	29 (5.1)	5 (7.5)	0.407

ICU: intensive care unit; ARDS: acute respiratory distress syndrome; AKI: acute kidney injury

### Comments

Aortic arch surgery requires transient cerebral circulatory arrest with or without hypothermia, leading to inevitable ischemic injury in the brain or organs [8, 9]. The procedure for TAR is more complicated compared with that of a hemiarch repair. Therefore, TAR is associated with higher incidences of death and neurological adverse events (NAE) than those of a hemi-arch repair, especially in an older cohort. Thus, a hemiarch repair is the preferred surgical procedure in an older cohort with ATAAD. However, aortic arch involvement was associated with higher incidences of rupture and reintervention [10–13]. The present study showed similar incidences of in-hospital death and major complications between different age groups. This promising outcome suggested that the older cohort, specifically those above 70 years old, may benefit from several advantages of the TBSG technique.

First, the open arch technique could facilitate the implantation of TBSG. All the arch vessel orifices and true lumen of the descending aorta could be easily identified through the arch incision, which resulted in fast and safe implantation of the TBSG. Therefore, the duration of circulatory arrest was short. Second, the short duration of the TBSG implantation allowed for TAR to be performed under moderate hypothermia circulatory arrest, thus, avoiding the potential risk of bleeding and inflammatory response caused by deep hypothermia circulatory arrest. Third, bilateral cerebral perfusion was possible, which provided more physiological and effective cerebral protection during circulatory arrest, thus, the circle of Willis does not need to be considered in this case. Furthermore, TAR only required a single anastomose, resulting in less branched vessels manipulation and reduced the difficulty of achieving hemostasis, which theoretically decreased the incidences of NAE and anastomotic oozing.

Endoleak development might occur after a TBSG implantation, but the incidence was low. This satisfactory result can be attributed to various reasons. First, the diameter of the stented elephant trunk was 10–20% larger than that of the proximal descending aorta, which could effectively prevent retrograde flow from the descending aorta. Second, the main graft portion was anastomosed to the native arch, which serves as the neo-intima of the arch. Third, the proximal end of the main tube graft was directly anastomosed to the Dacron graft, preventing an endoleak from the proximal end of the main tube graft. Furthermore, the base of the brachiocephalic vessels was stitched to prevent the migration of the TBSG. Finally, banding at the bases of the arch vessels was routinely applied when size of the sidearm stent graft was smaller than that of the corresponding arch vessel, which effectively prevented retrograde endoleak from the arch vessels.

Previous studies have reported that organ malperfusion is an independent risk of mortality [14, 15]. The distal part of the TBSG served as a stented elephant trunk, which could secure the tear located in the descending aorta, reverse the perfusion of the true lumen, and induce thrombosis in the false lumen of the downstream descending aorta, resulting in reperfusion of the ischemic organs. Reperfusion of organs could improve the perioperative outcomes.

### Limitations

There are several limitations in this study. First, we defined the older cohort as age > 70 years old, and only a few cases were within this specified range. Moreover, there may be potential selection bias in patients aged 70 years old and above for aortic arch surgery. Second, the present study was a retrospective study from a single center that was not randomized. Future multicenter research is required to validate the results of this study.

## Conclusion

TBSG implantation for ATAAD resulted in an acceptable mortality rate in older patients, specifically those above 70 years old, thus, it could be a feasible surgical procedure to perform in older patients when a TAR is required.

## Data Availability

All data generated or analysed during this study are included in this published article.

## Abbreviations

ATAAD: Acute type A aortic dissection
TAR: Total arch reconstruction
TBSG: Triple-branched stent graft
CPB: Cardiopulmonary bypass
SCP: Selected cerebral perfusion
ICU: Intensive care unit
NAE: Neurological adverse events
